# Feasibility and safety of Stanford A aortic dissection complete endovascular repair system in a porcine model

**DOI:** 10.1186/s12872-023-03494-3

**Published:** 2023-09-13

**Authors:** Yucheng Peng, Wenhui Lin, Deda Lou, Songyuan Luo, Bo Li, Mingcheng Su, Jitao Liu, Yue Tang, Jianfang Luo

**Affiliations:** 1 Foshan Fosun Chancheng Hospital, 3 Sanyou South Road, Chancheng District, Foshan, China; 2 Department of Cardiology, Guangdong Cardiovascular Institute, Guangdong Provincial People’s Hospital (Guangdong Academy of Medical Sciences), Southern Medical University, Guangzhou, China; 3 Chuangxin Medical Technology CO.Ltd, Shenzhen, China; 4 Cardiovascular Center, The Seventh Affiliated Hospital, Sun Yat-sen University, Shenzhen, China

**Keywords:** Acute type a aortic dissection, Thoracic aortic endovascular repair, Transcatheter, Porcine model, BRIDGE

## Abstract

**Background:**

Acute type A aortic dissection (ATAAD) is a catastrophic disease with high morbidity and mortality. Although open surgery is still the gold standard for the treatment of ATAAD, some patients, with advanced age and multiple comorbidities, can only receive medical management alone. Nowadays, thoracic aortic endovascular repair (TEVAR) provides a potential treatment option for the patient with ATAAD, but traditional stent grafts (SGs), which are not designed for the ATAAD, are inapplicable to the unique anatomy of the aortic arch. Therefore, we innovatively created the BRIDGE system (Chuangxin Medical, Shenzhen, China), a complete endovascular reconstruction system designed to treat ATAAD. This study aimed to evaluate the feasibility and safety of the novel Stanford A aortic dissection complete endovascular reconstruction system in a porcine model.

**Method:**

The BRIDGE system consists of the type A stent system and the type C stent system. Between November 2020 and March 2021, three white swine were utilized in the study. The BRIDGE system was deployed via the transcatheter approach under angiographic guidance. The swine(n = 3) treated with our system were evaluated using angiography before sacrifice 1-month after implantation, which was followed by gross specimen evaluation and histological examination of harvested tissues.

**Result:**

The acute procedure success rate was 100% (3/3). The immediate post-procedural angiography showed that both type A SGs and type C SGs were deployed in satisfactory locations, with patency of the supra-aortic trunk and no endoleak. The cumulative mortality of 30-day was 0% without any adverse events. No device migration or leakage was observed angiographically, before sacrifice. The gross observation confirmed a type A SG covered part of the entry of anonyma. Favorable endothelialization, no thrombogenesis, and slight inflammatory infiltration of the tissues around the device were confirmed by microscopic examinations in all pigs.

**Conclusion:**

It was feasible and secure to use Stanford A aortic dissection complete endovascular reconstruction system to implement a transcatheter endovascular repair in a porcine model. With this novel system, treating acute type A aortic dissection may be more efficient and secure in human.

**Supplementary Information:**

The online version contains supplementary material available at 10.1186/s12872-023-03494-3.

## Introduction

Aortic dissection is the most common acute aortic syndrome, which can be classified into Standford type A dissection and Standford type B dissection based on whether the ascending aorta is involved, regardless of the site of tear and irrespective of the distal extent of dissection [1, 2]. Acute type A aortic dissection(ATAAD), a catastrophic disease with high morbidity and mortality, accounts for 66% of acute dissections [3].

Thoracic aortic endovascular repair (TEVAR) is recommended by the current guidelines for treating patients with acute type B aortic dissection [4]. But open surgery is still the gold standard for treating ATAAD [4, 5]. Despite progress in the operative approach, median sternotomy, cardiopulmonary bypass, and hypothermic circulatory arrest could not be averted by the high-risk patients, and surgical mortality(18%) remain high yet [6–8]. Due to their advanced age and multiple comorbidities, some patients can only receive drug therapy alone with in-hospital mortality rates of 59% [8, 9].

A potential treatment option is provided by TEVAR in a subset of patients with ATAAD at high surgical risk but with suitable anatomy. Several studies reported favorable early and midterm results of TEVAR for patients with ATAAD. Nienaber et al. treated 12 patients with ATAAD with the proximal entry tear located between the coronaries and brachiocephalic artery with TEVAR, and the success rates of TEVAR and the 3-year survival rates are favorable (91.7% and 63.6%, respectively) [10]. Several studies reported patients with ATAAD, applied TEVAR, with 5-year survival rates of > 75% [11–13] In general most of the investigators selected off-the-shelf endograft components which were designed for the thoracic or abdominal aorta, and a small number of investigators would use a device designed for the ascending aorta (Cook Zenith Ascend Endograft) which could only cover the ascending aorta [14–16]. However, a study reported that only 9.4% of the dissections were confined entirely to the ascending aorta, and the majority would extend to the descending thoracic aorta (3.8%), abdominal aorta(11.3%), and beyond(52.3%) [17]. It suggested that the current devices can not meet the needs of the patient with ATAAD.

There is an urgent need to develop specialized devices for the endovascular treatment of ATAAD. We innovatively conceived and designed a BRIDGE system (Chuangxin Medical, Shenzhen, China) for treating ATTAD and assessed its feasibility and safety in a porcine model.

## Materials and methods

### Device system

**Fig. 1:**
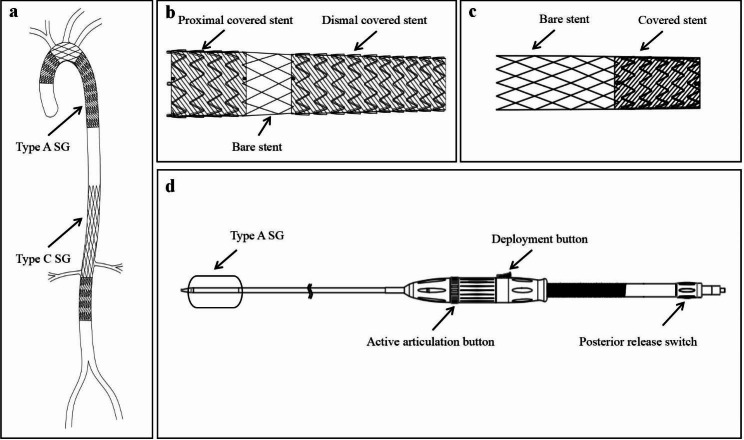
Design of BRIDGE system. The BRIDGE system consists of a type A stent system and a type C stent system. The type A SGs were deployed in the thoracic aorta, and the type C SGs were implanted in the abdominal aorta (**A**), the type A SG was comprised of covered stent segments in the two ends and a bare stent segment in the middle (**B**), the type C SG was comprised by a covered stent segment and a bare stent segment (**C**), the delivery system of type A SG allowed active articulation and posterior release (**D**)

The BRIDGE system consists of 2 devices:(1) a type A stent system ;(2) a type C stent system (Fig. 1). The type A stent system contains a type A stent graft (SG)and a delivery system. According to the characteristic anatomy of the swine’s aorta, the type A SG(CX2822 P40 M70 D160)was selected, which was 270 mm long and 5-10% increase beyond the maximal diameter of thoracic ascending aorta and thoracic descending aorta in the proximal and distal end. The type A SG was comprised of covered proximal and distal stents which are composed of a nitinol stent framework sutured to the outside of the polyester graft material, and a woven bare nitinol stent in between. The covered stent segments, designed to be implanted into the ascending aorta and the descending thoracic aorta, are used to cover the entry tear and re-expand the true lumen. The bare stent segment, arranged to be placed into the aortic arch, is further to re-establish true luminal blood flow and preserve the supra-aortic vessel patency simultaneously. The bare stent segment in type A SG was fabricated using 3-dimensional woven technology, which was able to provide the strength in both longitudinal and transverse directions. Consequently, type A SG provided a smoother surface when compared to the traditional stent. Furthermore, the bare stent segment of type A SG was made of nickel-titanium alloy in a braided pattern, with the “mesh” area of the stent ranging from 50 to 100 square millimeters. The delivery device includes a delivery handle with an articulation button, a delivery sheath, and a delivery pipe. The type A SG is delivered via a flexible delivery system that allows active articulation, and stable deployment.

**Fig. 2:**
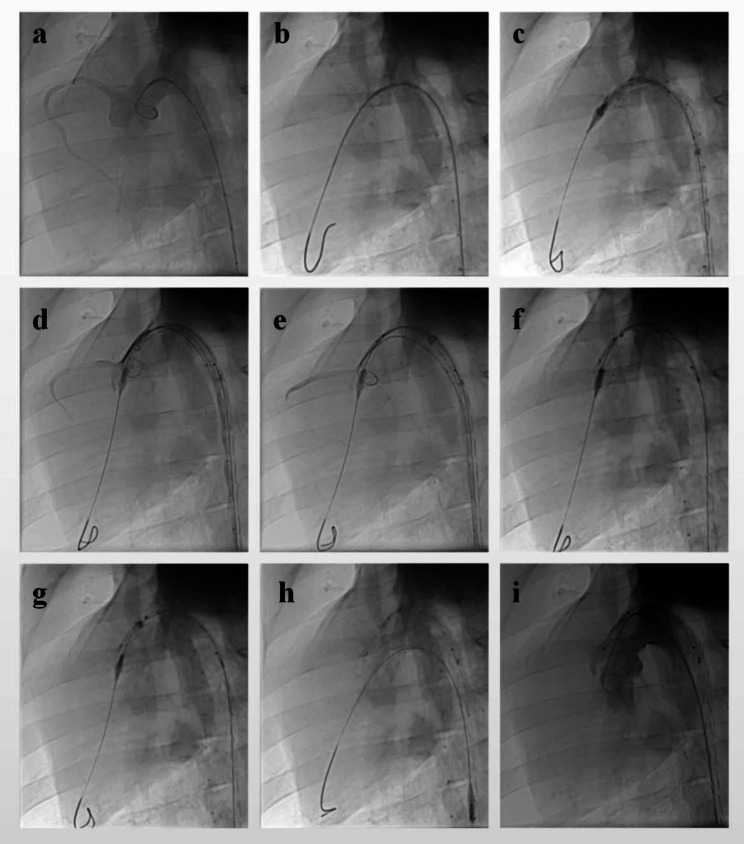
The procedure of BRIDGE system implantation. A pigtail catheter was placed in the right femoral artery for angiography (**A**), the stiff wire was placed in the left ventricle (**B**), the delivery sheath was inserted over a stiff wire and advanced to the ascending aorta (**C**), the proximal covered SG segment was deployed (**D**), the bare SG segment was released out (**E**), the distal covered SG segment can be deployed quickly (**F**), the posterior release wad performed (**G**), the delivery sheath of type A SG was retracted (**H**), a second angiography was performed (**I**)

The type C stent system includes a type C SG and a general delivery system. The type C SG, designed to be implanted into the descending aorta, includes a covered distal stent segment and a bare proximal stent segment(Fig. 2). The covered stent segment is constructed with a woven polyester fabric sewn to a self-expanding nitinol frame for sealing the dissected aorta and expanding the true lumen. The covered stent segments of both type A SG and type C SG abandoned the connecting bar, which was thought to create a greater spring-back force or SG-aorta interaction against the aortic wall, but was made of highly elastic nickel-titanium alloy and employed a design of supporting ring. The bare nitinol stent segment can be used to reshape the true lumen, avoid migration and preserve the visceral vessel patency simultaneously.

### Procedure

Between November 2020 and March 2021, three swine (Shanghai White pigs) were utilized for the experiment. All of the swine were male, and the mean weight was 75.0 ± 2.5 kg. Clopidogrel (75 mg per day) and aspirin (100 mg per day) was administered orally 3days before operation until the operation was performed. All the operators in the experiment worked extensively on the anatomy of cardiovascular system, and practiced for releasing of SG in vitro aortic simulation model via the delivery system (Supplement. Figure 1). The animal experiment was approved by the Guangdong Provincial People’s Hospital Ethics Committee (NO. GDREC2018215H(R3)).

The system was implanted under general anesthesia. Injecting intramuscularly azaperone (2 mg/kg) and ketamine (50 mg/kg) was for induction anesthesia, and isoflurane was for the maintenance of anesthesia. The procedure was guided by angiography (Fig. 2).

The access site was femoral arteries which were accessed percutaneously via the Seldinger technique. Systemic heparinization (100IU/kg) was administered in pigs after arterial access was achieved. A marker pigtail catheter was placed in the right femoral artery for angiography. Preoperative angiography was performed through the pigtail catheter to measure the diameter of the thoracic aorta and ensure patency of the coronary and brachiocephalic arteries. The catheter was placed across the aortic valve. Subsequently, the stiff wire was placed in the left ventricle. The delivery sheath was inserted over a stiff wire and advanced to the ascending aorta. The type A stent graft was preassembled on the tip of the delivery sheath. The proximal covered SG segment was accurately deployed by gently rotating the release handle until the bare SG segment was released out of the sheath. In the meanwhile, when the proximal covered SG segment was released, angiography was performed through the pigtail catheter to estimate the patency of the coronary artery. Before the bare stent segment was deployed, the proximal covered stent segment allows micro-readjustments so that the patency of both coronary and innominate arteries could be preserved. When the bare SG segment was released and a majority of SG was deployed stably, the distal covered SG segment can be deployed quickly by pressing down the quick release switch. Finally, the posterior release was performed by pulling down the posterior release switch. When the type A SGs were deployed in the thoracic aorta, a second angiography was performed to assess coronary arteries patency, arch vessel patency, and the presence of endoleak.

The delivery sheath of type A SGs was exchanged for a delivery sheath of type C SGs over a stiff wire. Angiography was performed again to guide the implantation of the type C SGs. The SGs could be adjusted to ensure that the entry of visceral vessels would not be covered before completion of the type C SGs deployment. When the type C SGs were implanted in the descending aorta completely, a completion angiogram was performed to assess the patency of the significant visceral vessel and the presence of migration. Finally, the pigtail catheter was retracted.

### Evaluation protocol

During the procedure, the rate of acute procedure success and complications were analyzed. After surgery, three swine were kept in a standardized animal room with daily injections of favalin 0.25 mg per day and aspirin 100 mg per day. Angiography was performed immediately after implantation and before sacrifice. Three pigs were euthanized utilizing a lethal does of potassium chloride 1-month after implantation. The thoracic aorta was then exposed through median sternotomy, and the whole heart, thoracic aorta and abdominal aorta were removed en bloc. A macroscopic examination of the excised aorta was performed and inspected to assess the SGs placement, tissue damage, device surface endothelialization, thrombosis and hemorrhage. The specimen was fixed in a 4% formalin solution, and 5 mm cross-sections were obtained at surrounding tissues of the proximal end, middle and dismal end of the implantation. Then the cross-sections were embedded in paraffin and stained with hematoxylin and eosin. Finally, these sections were examined under light microscopy to evaluate inflammatory infiltration, tissue damage, device surface endothelialization, thrombosis, and hemorrhage.

### Statistical analysis

Continuous variables were expressed as mean ± SD. Categorical variables were expressed as frequencies and percentages. Paired t-test was used for the comparison of continuous variables at different time points. Non-parametric Friedman test was used for the comparison of semi-quantitative Pathological changes results at different time points. All P values were two-tailed distributions, and P values < 0.05 were considered statistically significant. Statistical analysis was performed using the software SPSS version 19 (SPSS Inc., Chicago, IL).

## Result

### The acute results

**Fig. 3:**
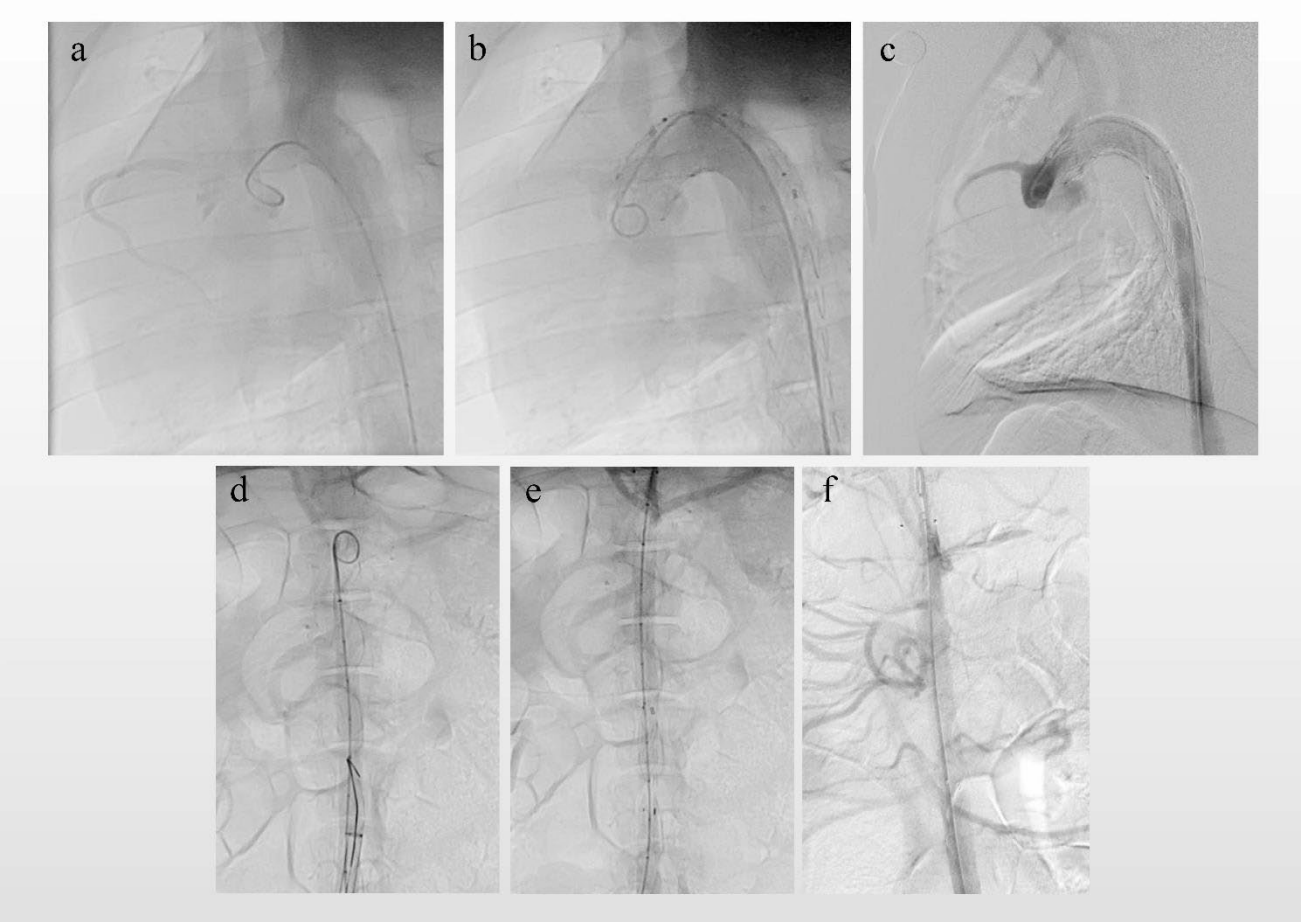
Angiography results of the thoracic aorta before device implantation (**A**), immediately (**B**), 30 days (**C**) after device implantation, and Angiography results of the abdominal aorta before device implantation (**D**), immediately (**E**), 30 days (**F**) after device implantation

A total of 3 pigs were used in this experiment. The characteristics of the pigs and the size of type A SG were listed in Table 1. All three pigs were male, and their mean weight was 75 ± 5 kg. Before the BRIDGE system was implanted, angiography showed that thoracic aorta and abdominal aorta were normal without any damage in three pigs (Fig. 3a, d). The acute procedure success rate was 100%(3/3). The Acute procedural success was defined in that both the type A and type B SGs were deployed in the appropriate location (while with patency of the supra-aortic trunk and the coronary artery), and successful withdrawal of the delivery system. Angiography immediately after the procedure demonstrated that both the type A and type C SGs were positioned in the appropriate location with the patency of the coronary arteries, the supra-aortic trunk and the significant visceral vessel in the other pigs. No endoleak was revealed by the angiography (Fig. 3b, e).

**Table 1:** The characteristics of the pigs and the size of type A SGs that were implanted in the pigs

Case No	Weight (Kg)	sex	Fellow-up (Month)	Diameter of proximal part of type A SGs, (mm)	Diameter of distal part of type A SGs, (mm)
1	72	male	3	28	22
2	80	male	3	28	22
3	73	male	3	28	22

Abbreviations: Kg, kilogram; mm, milimeter; SGs, sent grafts

### Follow-up results

Among the 3 pigs enrolled in this study, 3 pigs were sacrificed at 3-month after the procedure. The living habits, weight, and diet of all the pigs did not change significantly before the sacrifice. Angiography was performed on the pigs before sacrifice. Angiography 1-month after the procedure showed that type A and type C SGs were positioned in the satisfactory location in the three pigs, with the patency of the coronary arteries, the supra-aortic trunk and the significant visceral vessel. No endoleak, migration or deterioration of the SGs were observed in the angiography. Additionally, no aortoclasia, aortic aneurysm and aortic dissection can be revealed around the SGs by the angiography, indicating the excellent safety of the novel device (Fig. 3c, f).

**Fig. 4:**
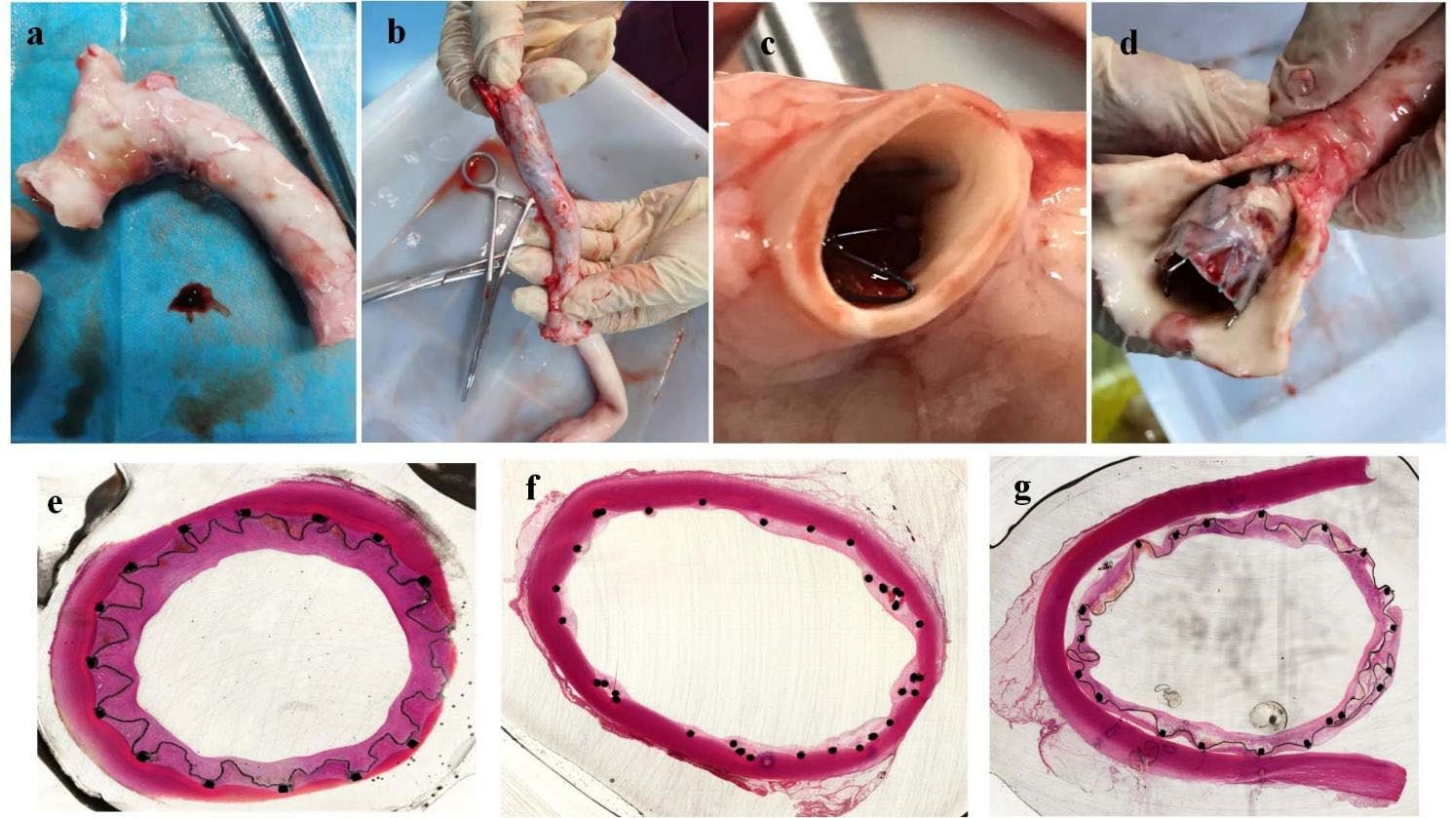
The Macroscopic examination of the thoracic aorta (**A-C**) and abdominal aorta (**D**) at 30 days after device implantation. Microscopic examination of the thoracic aorta in the proximal end (**A**), middle (**B**), and dismal end (**C**)

Macroscopic examination and Microscopic examination were performed after euthanasia in three pigs (Fig. 4). The gross observation confirmed that except for a type A SGs covered part of the innominate artery, other SGs were placed in the prospective location. All the SGs were embedded in the intima. Migration and breakdown of the SGs can not be observed. In addition, thrombus can not be found on the surface of the SGs in all the pigs.

Microscopic examination showed that both the type A and type C SGs were covered by endothelial cells with no thrombus. Slight inflammation infiltration and hemorrhage can be observed at surrounding tissues of the proximal end, middle and dismal end of the implantation. A slight increase in the trauma of the vessel wall can be found in surrounding tissues of type C SGs when comparing histological examinations were performed in the surrounding tissues of type A SGs and of type C SGs. And the trauma was mainly placed in the intima of the aorta. There was no obvious thrombosis、infarct or hemorrhage in the heart, liver, spleen, kidneys and brain of all the 3 pigs.

## Discussion

The BRIDGE system is a novel complete endovascular repair system aimed at treating ATAAD. The present study was designed to evaluate the feasibility and safety of this novel system in a porcine model. The type A and type C SGs were implanted in satisfactory locations with the convenient delivery system, and no procedure failure appeared in any of the 3 pigs. All the 3 pigs lived to the endpoint without any major complications. Angiography immediately, 1-month after implantation showed that both the type A and type C SGs were placed in the appropriate location with the patency of the coronary arteries, the supra-aortic trunk and the significant visceral vessel in the three pigs. However gross observation confirmed that a type A SGs covered part of the innominate artery. The partial coverage of the innominate artery in type A SGs may be caused by the rude retraction of the pigtail catheter during angiography 1-month after the procedure. When the pigtail catheter was attached to the type A SGs, rude retraction led to the migration of the SGs, and the innominate artery was covered partially. To avoid this unpleasant situation, the catheter should be retracted mildly. But the partial coverage of the innominate artery is considered acceptable, because angiography showed that there is no flow limiting in the innominate artery. Macroscopic and Microscopic examination confirmed that all the SGs showed excellent endothelialization on the surface, indicating the suitable biocompatibility of the SGs. Slight inflammation infiltration and hemorrhage were revealed at the surrounding tissues of SGs by the microscopic examination, but no thrombus was observed. During the procedure, insufficient asepsis should be responsible for the inflammation infiltration and hemorrhage. Additionally, a slight increase in trauma of vessel wall was found in the surrounding tissues of type C SGs. Some oversized type C SCs, which lead to the large radial force, explicate the trauma of the vessel wall observed at surrounding tissues of type C SGs. Therefore, to select a suitable size of the SGs, measurement of the length and diameter of the aorta with a gated computed tomography scan before the procedure was necessary.

Nowadays, Open surgery is still considered to be the gold standard in treatment for ATAAD, but its mortality and perioperative complications rate remain high especially when high-risk patients and elderly patients are taken into consideration [6]. TEVAR has emerged as a viable and potentially suitable alternative for patients who have prohibitive surgical risks and are treated with medical management with subsequent high mortality. Since Dorros et al. performed the first successful endovascular repair in a patient with an ATAAD in 2000, the endovascular treatment of ATAAD was proposed as an alternative to open surgery in very selected high-risk or inoperable patients, with satisfactory preliminary results [18, 19]. In recent years, to improve the outcome, several complete endovascular techniques, including chimney SG, branched SG and fenestration, are used in addition to the TEVAR [20]. These combined techniques have contributed to the development of the complete endovascular technique for the treatment of complex arch anatomies [21–23]. However, the disadvantage and weakness of these techniques should be responsible for the unsatisfactory prognosis and reintervention. For the fenestrated devices, the failure of aligning the fenestration with the orifice of the branch artery may induce the coverage of the supra-aortic trunk. When the chimney SGs were used, they may increase the risk of endoleaks after the procedure [24]. Branched-type stent grafts, which are customized, could not be available in emergency cases due to the diversity in the three branches of the aortic arch, especially for elderly patients with concomitant diseases [25].

To solve these problems, the BRIDGE system would be an important technical tool. In our system, the type A SG is placed primarily in the thoracic aorta, with the membrane stents in the ascending aorta and descending aorta, and a bare stent in the aortic arch. The membrane stents can cover the rupture of the aortic dissection, reshape the true lumen and promote thrombogenesis in the false lumen. The type C SG is mainly placed in the abdominal aorta, and the membrane stent can also cover the breach and open re-shape the true lumen, while the proximal bare stent can further re-shape the aorta and strengthen the stability of the stent without covering the important branches of the abdominal aorta, such as the renal artery. Compared with off-the-shelf versions, designed for the descending thoracic aorta or abdominal aorta originally, type A SG showed sufficient length and distinctive structure, which would improve its stability and decrease the risk for stent migration. Furthermore, the sufficient length of the type A SG could provide a smaller anchoring zone than before, which could reduce the risk for occlusion of the coronary artery. In addition to the SG, improvements have been made to the delivery system in the BRIDGE system as well. The type A SG was delivered via a flexible delivery device that allows active articulation, stable deployment and recapturing if needed. Due to the active articulation and soft catheter tip of type A SG, delivery catheter was able to avoid contacting with aortic wall and reduce the risk for the aortic arch rupture during crossing of the arch. The improvements from stent to delivery system were all geared towards administering safer and more effective treatments for type A aortic dissection than before.

In this study, postoperative angiography and anatomy results showed that the BRIDGE system could be successfully implanted into the aorta. Based on the previous roles of the membrane stent in closing the aortic dissection breach, reshaping the aorta, and promoting thrombogenesis, it can be inferred that the new system can be used in the treatment of acute type A aortic dissection. Meanwhile, the pigs with implanted stents all survived healthily until the study’s end time. Based on the results of the 30-day postoperative follow-ups, no displacement or deformation of the stents of Stanford A aortic dissection complete endovascular reconstruction system occurred after implantation. The aorta implanted with the system was largely free of rupture, dilatation, and other abnormalities, and no adverse conditions such as thrombosis, severe inflammatory cell infiltration, hemorrhage, or ischemia were observed in pathological sections. The findings confirmed the short-term safety of the BRIDGE system in porcine models.

There are two main limitations of this experiment. First, because a porcine model of aortic dissection has not yet been stably constructed, experiments with this new system could only be performed on healthy pigs. Second, the follow-up period for this experiment was only 90 days, and in fact, a longer follow-up period could be conducted later to determine the impact of the system. In addition, the trial was conducted on animals, and a clinical trial is needed to more fully estimate the safety and feasibility of the system.

## Conclusion

The BRIDGE system is a maneuverable and functional device with satisfactory early results in the porcine model. This system may provide a more effective and secure treatment for the patient with type A aortic dissection, especially the aged with lots of complications. Long-time follow-up and clinical trials are needed to verify the effect of the Stanford A aortic dissection complete endovascular reconstruction system for treating ATAAD in human.

### Electronic supplementary material

Below is the link to the electronic supplementary material.

Supplementary Material 1

## Data Availability

The data collected and analyzed during the current study are available from the corresponding author on reasonable request.
