# Correction: Feasibility and safety of Stanford A aortic dissection complete endovascular repair system in a porcine mode

**DOI:** 10.1186/s12872-023-03546-8

**Published:** 2023-10-24

**Authors:** Yucheng Peng, Wenhui Lin, Deda Lou, Songyuan Luo, Bo Li, Mingcheng Su, Jitao Liu, Yue Tang, Jianfang Luo

**Affiliations:** 1 Foshan Fosun Chancheng Hospital, 3 Sanyou South Road, Chancheng District, Foshan, China; 2 Department of Cardiology, Guangdong Cardiovascular Institute, Guangdong Provincial People’s Hospital (Guangdong Academy of Medical Sciences), Southern Medical University, Guangzhou, China; 3 Chuangxin Medical Technology CO.Ltd, Shenzhen, China; 4 Cardiovascular Center, The Seventh Affiliated Hospital, Sun Yat-sen University, Shenzhen, China

**Correction to: BMC Cardiovascular Disorders (2023) 23:455**

10.1186/s12872-023-03494-3

In this article [1], the grant number in the funding section was missing from this article and should have read “The author(s) disclosed receipt of the following financial support for the research, authorship, and/or publication of this article: This research was supported by National Natural Science Foundation of China (82200519); Natural Science Foundation of Guangdong Province, China (2022A1515010897) and Medical Scientific Research Foundation of Guangdong Province, China (A2021348). The funding bodies did not have any role in the design of the study, data collection, and analysis, nor on the interpretation and dissemination of the results.”

The original article has been corrected.
