# Supplementary material for: Feasibility and safety of Stanford A aortic dissection complete endovascular repair system in a porcine model
Source: BMC Cardiovasc Disord. 2023 Sep 13;23:455. doi: 10.1186/s12872-023-03494-3 (PMC10500764; doi:10.1186/s12872-023-03494-3)

Supplement. Figure 1. The type A SG and type C SG which were released in vitro aortic simulation model.


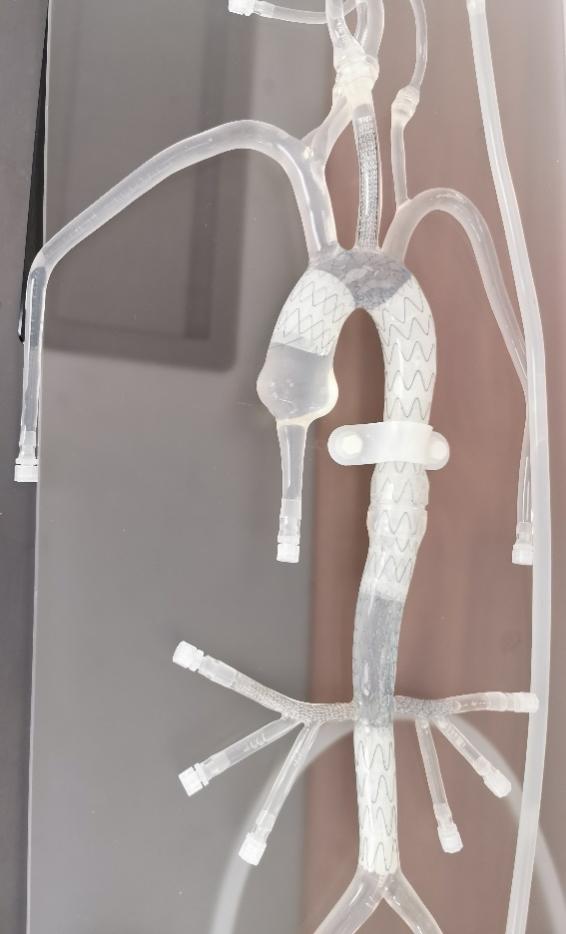

Supplement: Supplementary file 1 — Supplementary Material 1 [file 12872_2023_3494_MOESM1_ESM.docx]
